# Supplementary material for: Chemical reactivity of RNA and its modifications with hydrazine
Source: Commun Chem. 2025 Feb 14;8:48. doi: 10.1038/s42004-025-01444-y (PMC11829040; doi:10.1038/s42004-025-01444-y)
Supplement: Supplementary file 2 — Supplmentary Information [file 42004_2025_1444_MOESM2_ESM.pdf]

Supplementary Data

Chemical reactivity of RNA and its modifications with hydrazine

Nur Yeşiltaç-Tosun[1], Yuyang Qi[1], Chengkang Li[1], Helena Stafflinger[2], Katja Hollnagel[2], Leona Rusling[3], Jens Wöhnert[2], Steffen Kaiser[3], Stefanie Kaiser[1,*]

[1]Institute of Pharmaceutical Chemistry, Goethe-University Frankfurt, Max-von-Laue-Str. 9, 60438 Frankfurt/M., Germany

[2]Institute for Molecular Biosciences, Goethe-University Frankfurt, Max-von-Laue-Str. 9, 60438 Frankfurt/M., Germany

*Corresponding author: stefanie.kaiser@pharmchem.uni-frankfurt.de

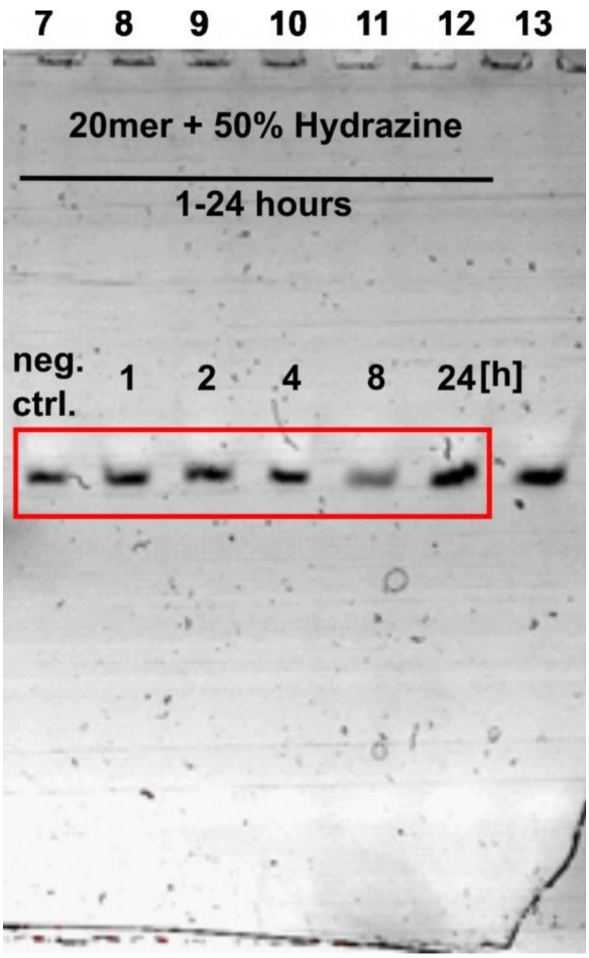

Fig. S1 20% TBE-urea polyacrylamide-gel of 20mer RNA exposed to 50% hydrazine up to 24 hours at 4°C (lane 7 to 12). The red box shows the intact RNA.

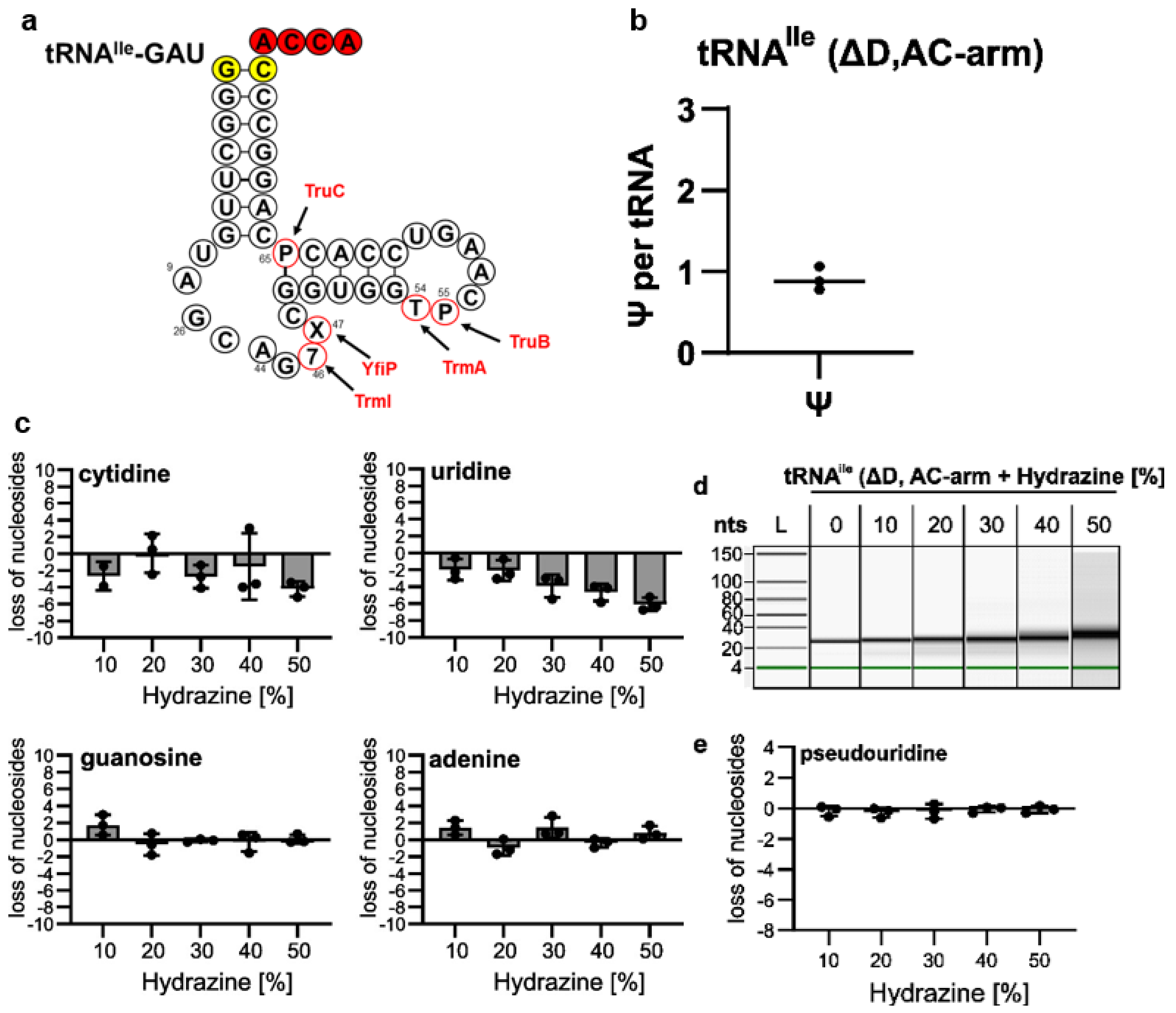

Fig. S2 Abundance of pseudouridine (Ψ) and canonical nucleosides in tRNA^{Ile} ΔD,AC-arm. (a) Sequence and potential fold of the mini-tRNA^{Ile} ΔD,AC-arm. **(b)** TruB efficiently installs Ψ in tRNA^{Ile} with deleted D-arm, quantified by LC-MS/MS. From n=3 biological replicates. **(c)** The abundance of canonical nucleosides in tRNA^{Ile} ΔD,AC-arm (TruB pre-incubated) was assessed by LC-MS/MS after treatment with 10%-50% hydrazine for 1 hour at 4 °C and over-night-precipitation by 0.1x 5M NH_4OAc and 2.5x ice-cold ethanol. **(d)** Bioanalyzer small-chip of hydrazine-treated tRNA^{Ile} ΔD,AC-arm. **(e)** The abundance of pseudouridine in tRNA^{Ile} ΔD,AC-arm was assessed by LC-MS/MS after treatment with 10%-50% hydrazine for 1 hour at 4 °C.

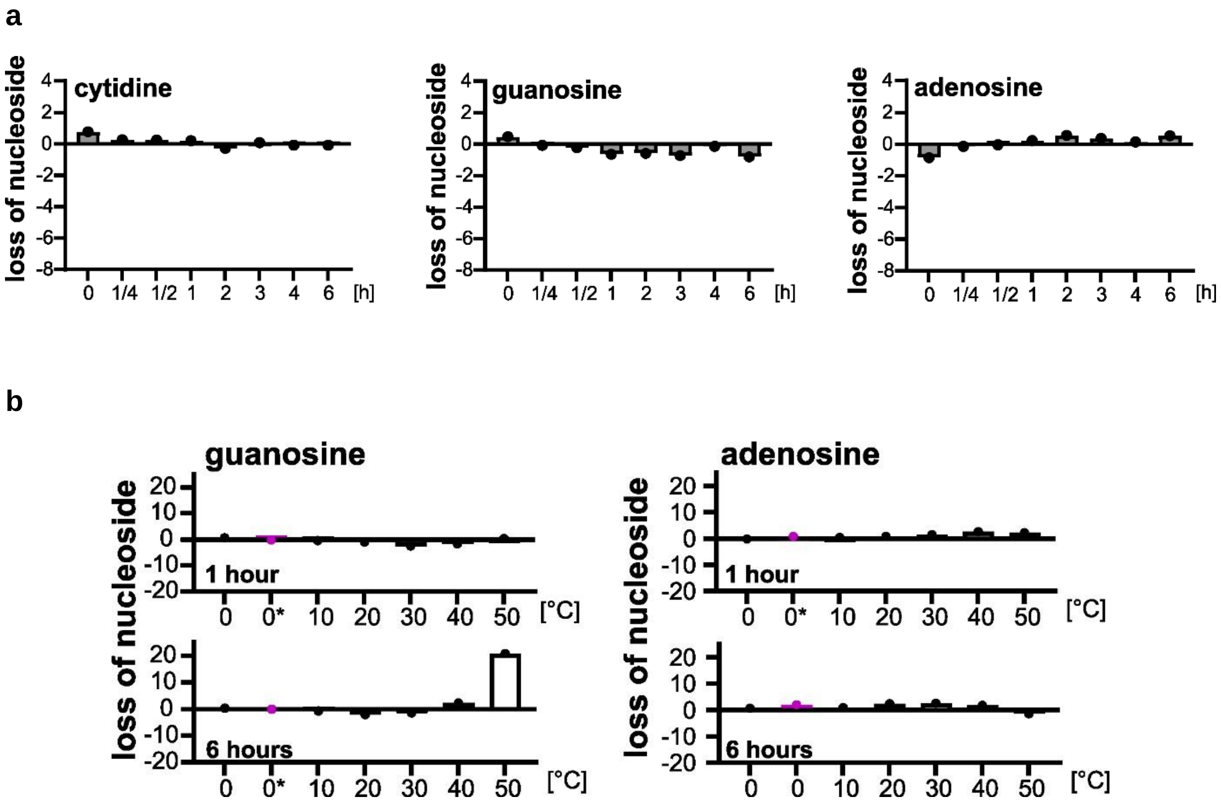

Fig. S3 Abundance of canonical nucleosides in $tRNA^{Ile}$ after hydrazine treatment. **(a)** Using 30% hydrazine incubation for 0.25-6 hours. **(b)** Using 30 % hydrazine at the indicated temperatures for 1 hour (top) or 6 hours (bottom). (0* = 50%)

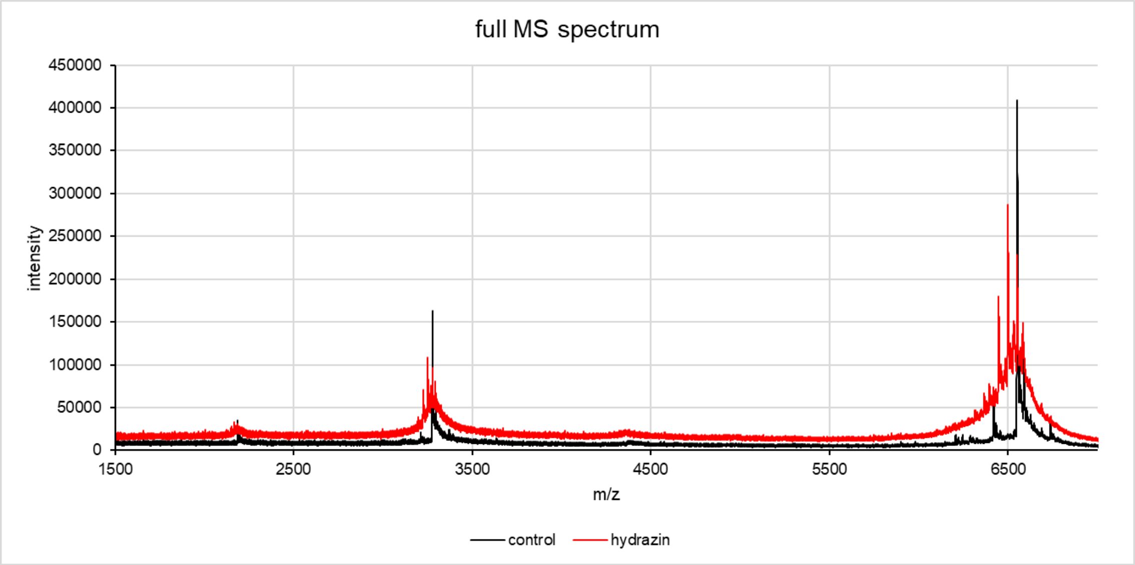

Fig. S4 Full spectrum of 20-mer before (control) and after treatment with 30% hydrazine at 0°C for 1 hour. Spectra were recorded in positive ion mode on a MALDI-TOF using 3-HPA.

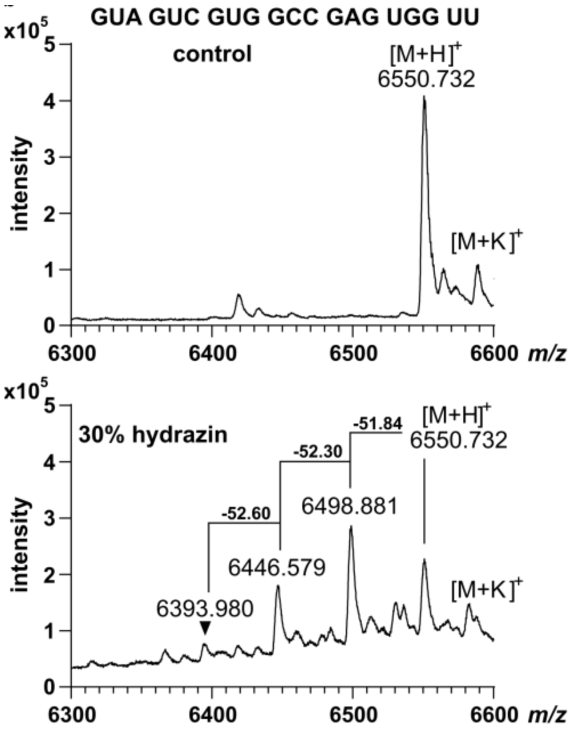

Fig. S5 Zoomed spectrum of 20-mer before (control) and after treatment with 30% hydrazine at 0°C for 1 hour. Spectra were recorded in positive ion mode on a MALDI-TOF using 3-HPA.

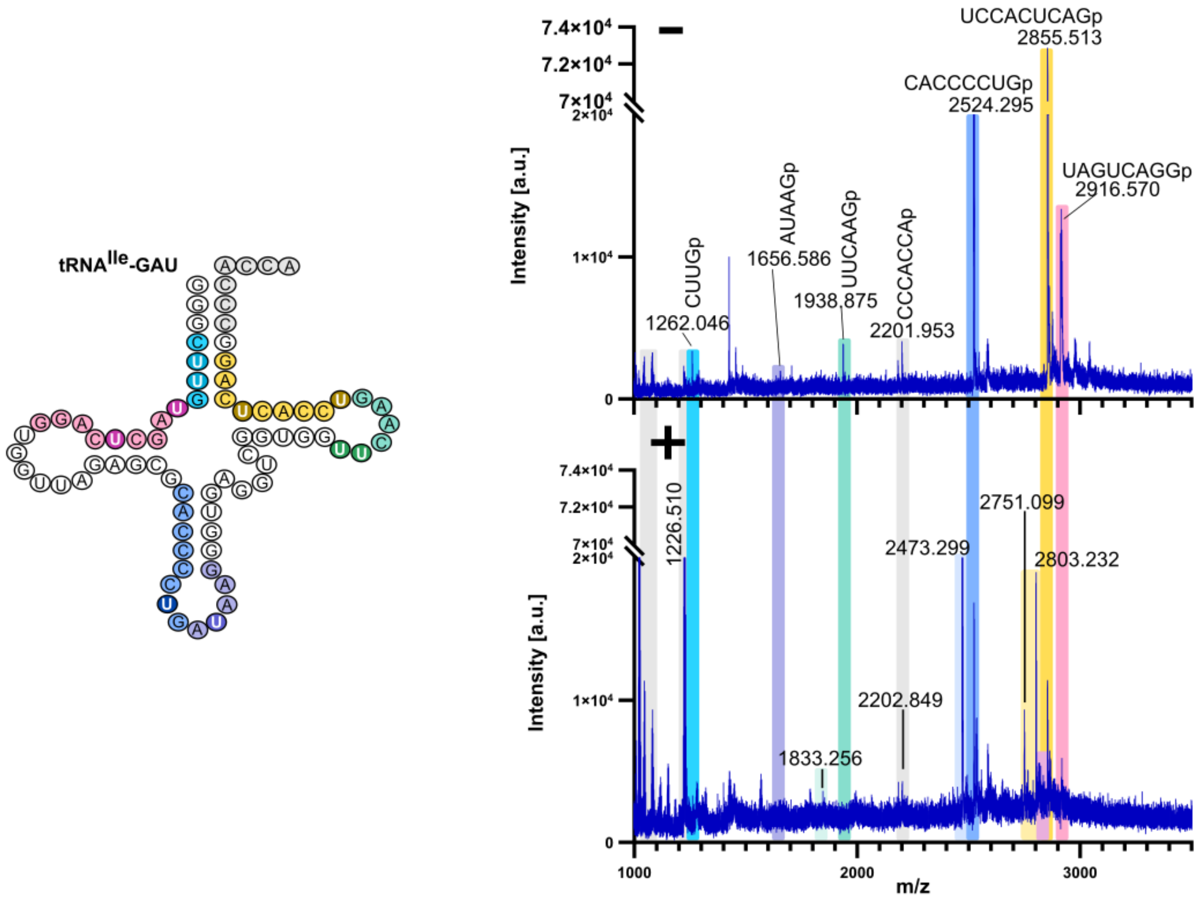

Fig. S6 Full spectrum of tRNA^{Ile} using RNase T1 hydrolysis. Top: before (control) and bottom after treatment with 50% hydrazine, at 4 °C for 6 hours. Detected oligonucleotides are color-coded in tRNA cloverleaf and in both spectra. The products after hydrazine treatment, are located 52 Th lower which is highlighted with slighter-colored boxes in the bottom spectrum.

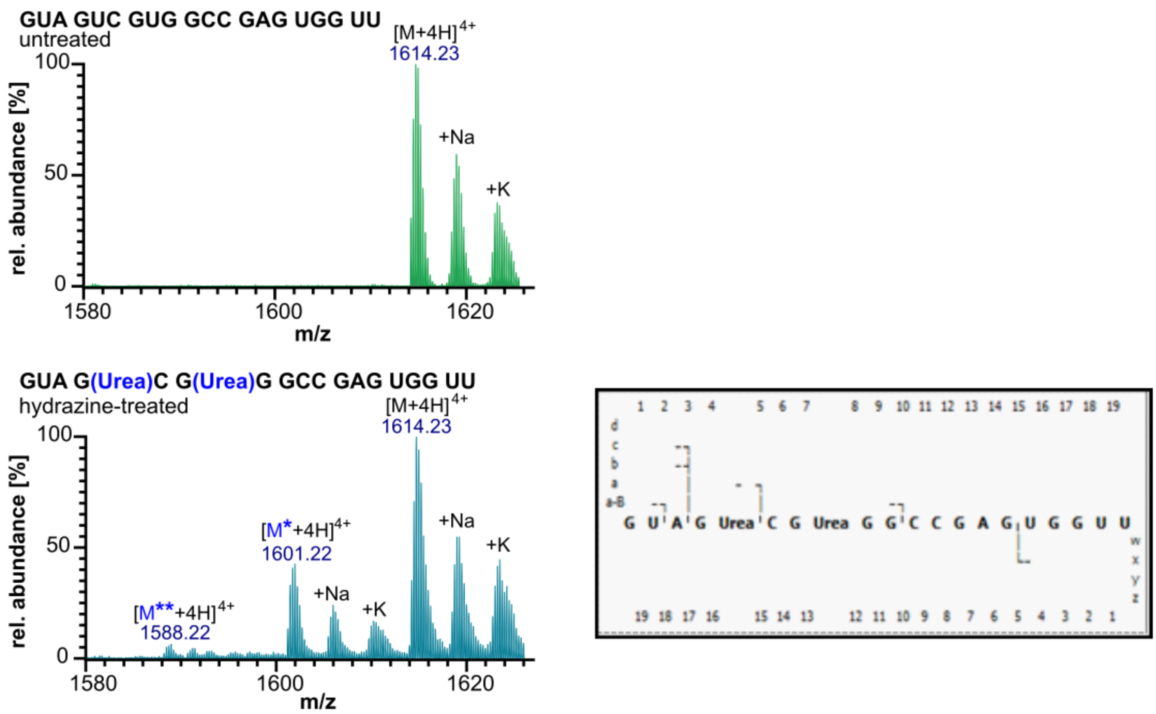

Fig. S7 Mapping of urea-ribose onto the 20-mer RNA. The 20-mer RNA was incubated with 20% hydrazine for 1 hour and subjected to high-resolution mass spectrometry. Analysis of the MS and MS/MS data by NASE (Wein et al.) revealed the location of 2 converted uridines as urea-ribose.

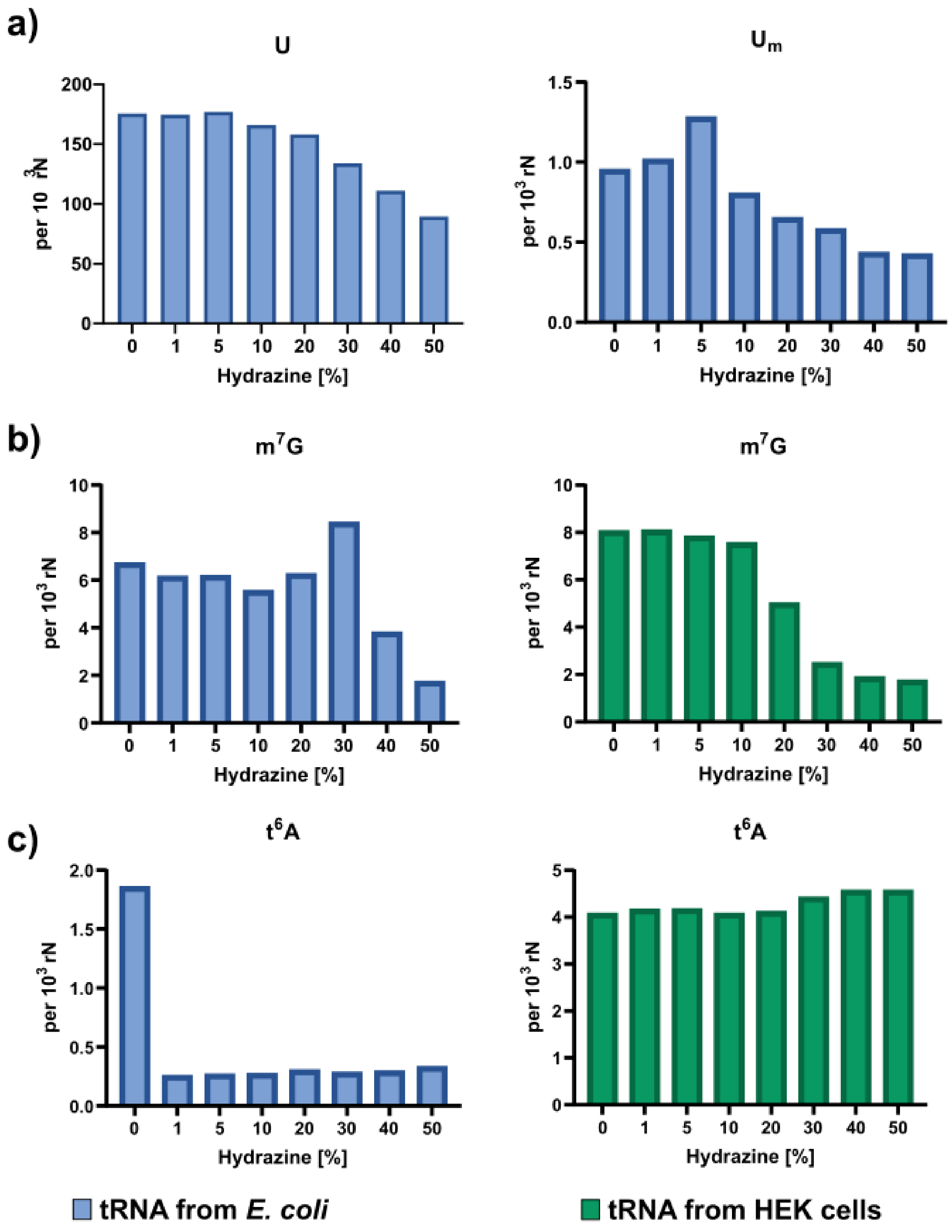

Fig S8 Absolute abundance of nucleosides after incubation with various hydrazine concentrations for 1 hour at 4 °C. (a) uridine (U) and 2'-O-methyluridine (Um). **(b)** N7-methylguanosine (m^7G) in E. coli and human tRNA. **(c)** N6-threonylcarbamoyladenosine (t^6A) in E. coli and human tRNA.

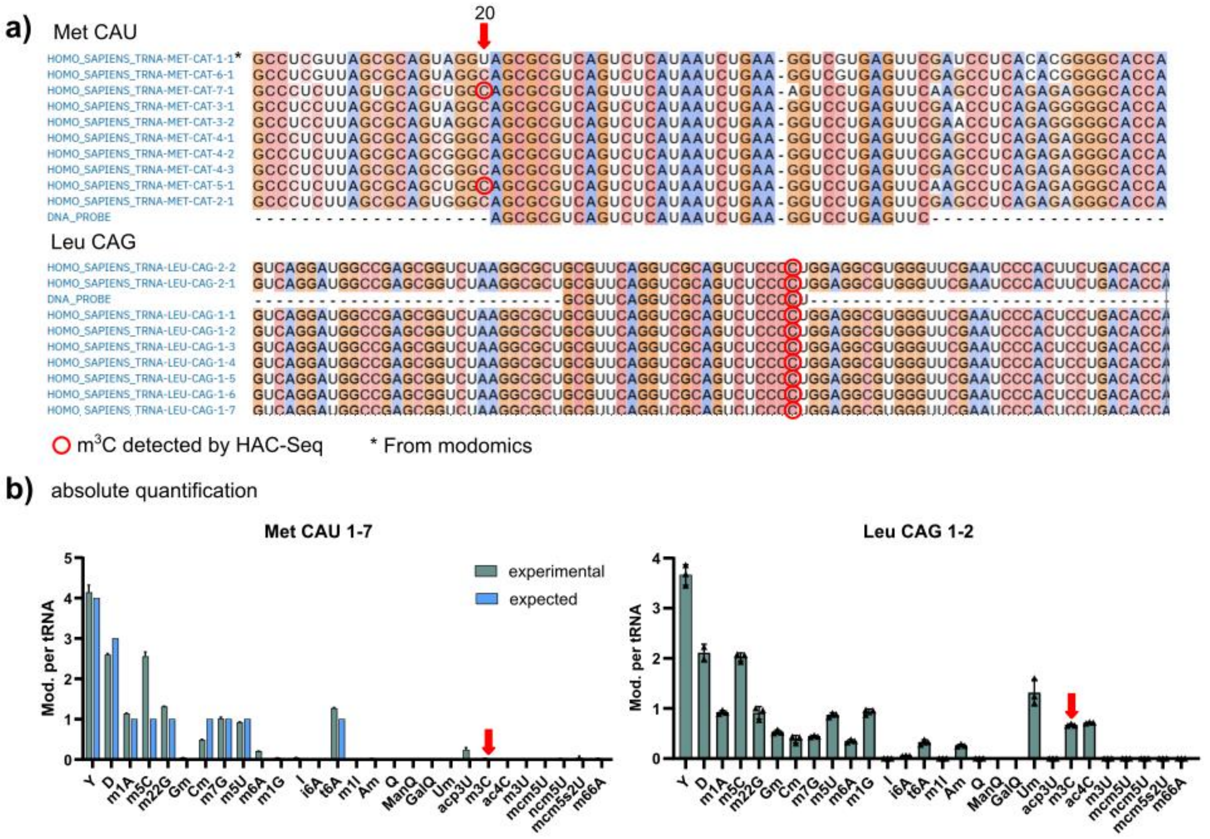

Figure S9 Quantitative mass spectrometry analysis of cytosolic tRNA Met-CAU and Leu-CAG from Homo sapiens. (a) Sequences of human cytosolic tRNA Met-CAU and Leu-CAG isoforms. An asterisk (*) denotes sequences reported in Modomics. Red circles indicate m^3C modification sites identified in the isoforms of tRNA Met-CAU and Leu-CAG. DNA probes specific to tRNA Met-CAU and Leu-CAG, designed to be reverse complementary to all corresponding isoforms, were used separately for purification and are aligned with the corresponding sequences. **(b)** Absolute quantification of modifications in tRNA Met-CAU and Leu-CAG. The modification profile of Met-CAU was compared to the reference data reported in Modomics.

Table S1: Products and high accuracy MS spectra of hydrazine reaction products			
**Nucleoside short name**	**product**	**MS1 spectra**	**Accuracy [ppm]**
acp^{3}U	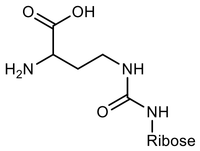 Theoretical mass: 294,12958	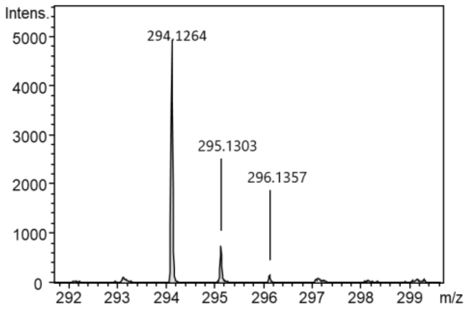	10.812
s^{2}C	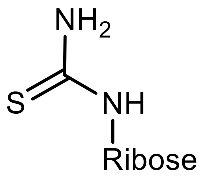	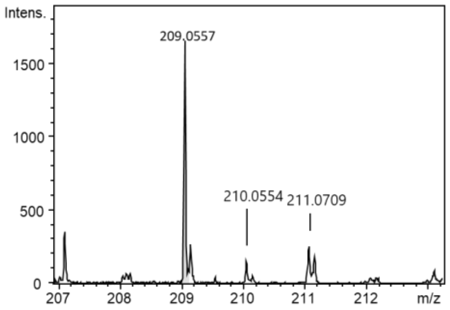	16.024
m^{3}C	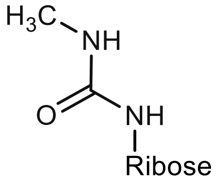	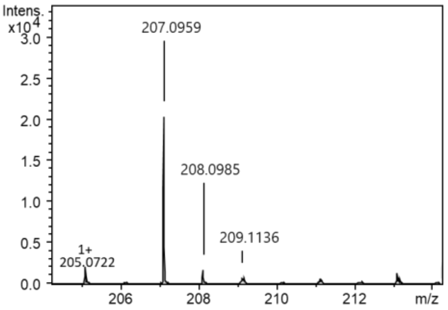	7.967

<table>
<tr><th colspan="3">Table S2: MS/MS spectra of products</th></tr>
<tr><th></th><th>MS/MS</th><th>MS³</th></tr>
<tr><td>acp^{3}U</td><td>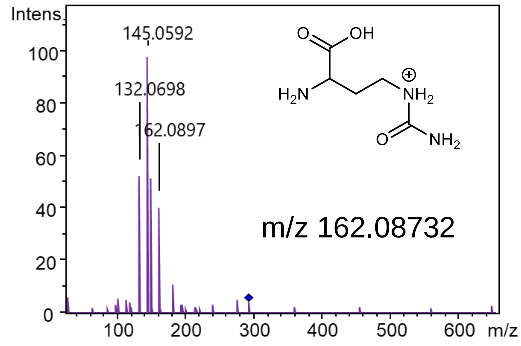
</td><td>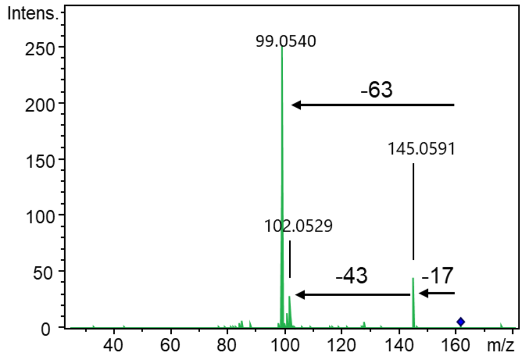
</td></tr>
<tr><td>s^{2}C</td><td colspan="2">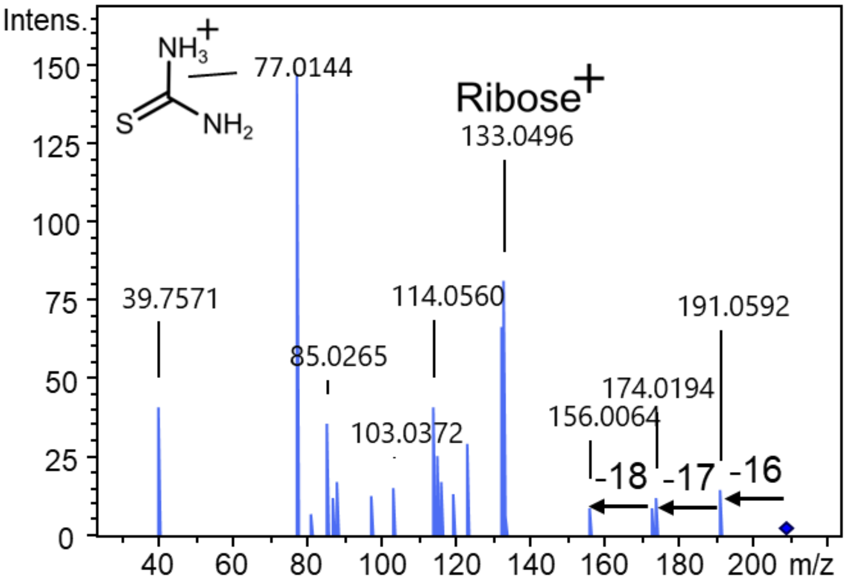
</td></tr>
<tr><td>m^{3}C</td><td colspan="2">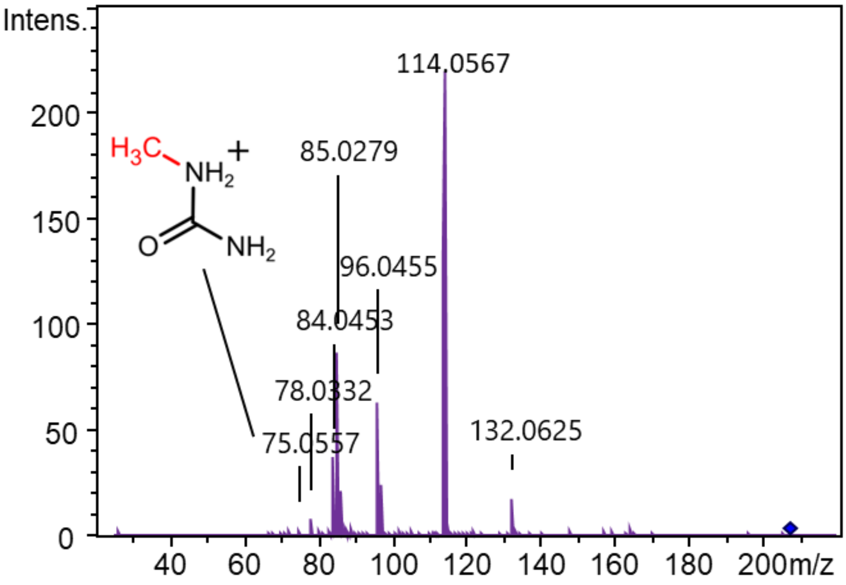
</td></tr>
</table>

Table S3. Accurate mass MS measurents of Nucleoside-Hydrazine reaction products**.** Elucidated molecular formulas, *m/z* calculations and results, collision energy (CE) and in-source collision induced dissociation (isCID) conditions.

Nucleoside (+ Hydrazine Product)	Molecular Formula	Ion Species	Theoretical *m/z*	Measured *m/z*	Mass Error (ppm)	CE (V)	isCID (V)
s^4U	$C_9H_{14}N_4O_5$	$[M+H]^+$	259.10370	259.1002	-13.51	25.00	0
s^4U Base	$C_4H_6N_4O$	$[M+H]^+$	127.06144	127.0609	-4.250	38.00	30.00
D	$C_9H_{18}N_4O_6$	$[M+H]^+$	279.12991	279.1291	-2.902	15.00	0
D Base	$C_4H_{10}N_4O_2$	$[M+H]^+$	147.08765	147.0884	5.099	15.00	20.00
m^7G (N7-methyl-FAPy-G)	$C_{11}H_{17}N_5O_6$	$[M+H]^+$	316.12516	316.1228	-7.465	17.10	0
m^7G Base	$C_6H_9N_5O_2$	$[M+H]^+$	184.08290	184.0822	-3.803	23.10	60.00
acp^3U	$C_{10}H_{19}N_3O_7$	$[M+H]^+$	294.12958	294.1264	-10.81	18.00	0
acp^3U Base	$C_5H_{11}N_3O_3$	$[M+H]^+$	162.08732	162.0850	-14.31	22.00	20.00
s^2C	$C_6H_{12}N_2O_4S$	$[M+H]^+$	209.05905	209.0557	-16.02	20.00	0
s^2C Base	CH_4N_2S	$[M+H]^+$	77.01680	77.0182	18.18	40.00	40.00
m^3C	$C_7H_{14}N_2O_5$	$[M+H]^+$	207.09755	207.0959	-7.967	20.00	0
m^3C Base	$C_2H_6N_2O$	$[M+H]^+$	75.05529	75.0563	13.46	N/A	N/A

Table S4. Accurate mass MS measurents of Nucleoside-Hydrazine reaction products**.** Bruker MicrOTOF-qII Acquisition parameters.

Source Parameters		Tune Parameters	
		Funnel 1 RF (Vpp)	200.0
End Plate Offset (V)	500	Funnel 2 RF (Vpp)	200.0
Capillary (V)	4500	Hexapole RF (Vpp)	100.0
Nebulizer (Bar)	1.0	Quadrupole Ion Energy (eV)	4.0
Dry Gas (L/min)	4.0	Collision RF (Vpp)	140.0
Dry Temperature (°C)	220	Collision Cell: Transfer Time (µs)	65.0
		Collision Cell: Pre-Pulse Storage (µs)	5.0
